# The protocol for the Families First Edmonton trial (FFE): a randomized community-based trial to compare four service integration approaches for families with low-income

**DOI:** 10.1186/1472-6963-14-223

**Published:** 2014-05-19

**Authors:** Jane Drummond, Laurie Schnirer, Sylvia So, Maria Mayan, Deanna L Williamson, Jeffrey Bisanz, Konrad Fassbender, Natasha Wiebe

**Affiliations:** 1 Faculty of Nursing, University of Alberta, Edmonton Clinic Health Academy, 11405 87 Avenue, Edmonton Alberta, AB T6G 1C9, Canada; 2 Faculty of Extension, University of Alberta, Edmonton, AB T5J 4P6, Canada; 3 Department of Human Ecology, Faculty of Agricultural, Life & Environmental Sciences, University of Alberta, Edmonton, AB T6G 2R3, Canada; 4 Department of Psychology, Faculty of Arts, University of Alberta, Edmonton, AB T6G 2E9, Canada; 5 Department of Oncology, Faculty of Medicine & Dentistry, University of Alberta, Edmonton, AB T6G 1C9, Canada; 6 Department of Medicine, Division of Nephrology, University of Alberta, Edmonton, AB T6G 1C9, Canada

**Keywords:** Low-income families, Service integration, Healthy families, Recreation, Pragmatic trial

## Abstract

**Background:**

Families with low incomes experience an array of health and social challenges that compromise their resilience and lead to negative family outcomes. Along with financial constraints, there are barriers associated with mental and physical health, poorer education and language. In addition, vulnerable populations experience many services as markedly unhelpful. This combination of family and service barriers results in reduced opportunities for effective, primary-level services and an increased use of more expensive secondary-level services (e.g., emergency room visits, child apprehensions, police involvement). A systematic review of effective interventions demonstrated that promotion of physical and mental health using existing service was critically important.

**Methods/Design:**

The Families First Edmonton Trial (FFE) tests four service integration approaches to increase use of available health and social services for families with low-income. It is a randomized, two-factor, single-blind, longitudinal effectiveness trial where low-income families (1168) were randomly assigned to receive either (1) Family Healthy Lifestyle plus Family Recreation service integration (Comprehensive), (2) Family Healthy Lifestyle service integration, (3) Family Recreation service integration, or (4) existing services. To be eligible families needed to be receiving one of five government income assistance programs. The trial was conducted in the City of Edmonton between January 2006 and August 2011. The families were followed for a total of three years of which interventional services were received for between 18 and 24 months. The primary outcome is the number of family linkages to health and social services as measured by a customized survey tool “Family Services Inventory”. Secondary outcomes include type and satisfaction with services, cost of services, family member health, and family functioning. Where possible, the measures for secondary outcomes were selected because of their standardization, the presence of published norming data, and their utility as comparators to other studies of low-income families. As an effectiveness trial, community and government partners participated in all committees through a mutually agreed upon governance model and helped manage and problem solve with researchers.

**Discussion:**

Modifications were made to the FFE trial based on the pragmatics of community-based trials.

**Trial registration number:**

ClinicalTrials.gov NCT00705328

## Background

A pervasive challenge faced by Canadian health and social service providers is to promote health for vulnerable populations in a proactive and cost-effective manner. Indeed, families with low incomes continue to experience an array of health and social barriers that compromise their resilience, leading to negative family outcomes, and inhibiting access to available services. The relation between low income and poor health is well known. In a recent systemic review, researchers found higher rates of mental health problems among children with low socioeconomic status [1]. The Canadian infant mortality rate is two-thirds higher in low-income neighbourhoods than in the highest income neighbourhoods [2]. Low-income Canadian children are more likely than their higher-income peers to suffer from ill health and mental illness [3], to be hospitalized [4], to have lower levels of cognitive scores and educational attainment [5], to have compromised memory and self-regulation [6], and to experience major behavioural problems [6-15]. Living with a low income compromises parental health and ability to parent, threatens family resilience, and jeopardizes family-community relations [16,17]. The health of low-income parents influences their ability to attain and maintain employment [18-23]. In addition, the constraint in public expenditure and commensurate movement away from universal services [24], which began in the 1990s, continues to affect those living with low incomes the most.

### Low-income and the social determinants of health

Based on decades of inquiry, the World Health Organization [25], the Public Health Agency of Canada [26], and the Canadian Medical Association [27] have agreed that health and well-being are largely shaped by social and economic contextual factors, which are collectively referred to as the “social determinants of health.” Contextual factors such as low educational attainment and low-wage precarious employment negatively influence the health of low-income families and their members by limiting their capacity to (a) increase their income and (b) obtain and use health and social services.

### Increasing income

Despite growth in the Canadian economy during the mid 2000s, which resulted in an increase in jobs and a decline in unemployment rates [28], the incidence of low income among Canadian families remains essentially unchanged [29,30]. Further, in Canada, having work does not preclude having a low income. For instance, in two-parent families with one earner, 23% have low incomes [31]. In female-headed single-parent families where the mother is employed, 43% are living on low incomes [32]. In Edmonton, the site of the study, 18.5% of families and 26.5% of children have low income [33]. Provincially, decision-makers are interested in providing services to low-income families that are both beneficial and cost effective [34] (e.g. Alberta’s Social policy framework [35] and the Results-based Budgeting initiative [36]).

Moving social assistance recipients from welfare to employment was a main goal of the 1990s social policy reform across Canada. In these programs low-income recipients of social assistance are required to get jobs or engage in employability programs [37-40]. Evaluative studies of welfare-to-work initiatives in Canada and the U.S. [21,22,41-44] suggest that policy efforts to move parents from social assistance to employment have been successful [22,42,44,45] but do little to ameliorate the negative health consequences of living on a low income. That is, welfare-to-work programs seem to influence parents’ health and children’s development only when increased parental employment is accompanied by increases to family income [17,22,41,44]. There are two ways to increase income in working families: increase income from employment and provide income supplements, such as the Self-Sufficiency Project [42,44]. The majority of parents who move from social assistance to employment plus income supplement continue to have family incomes below low-income cut-offs. Furthermore, their income tends to decline when supplements end [42,44,46]. Finally, the evidence is mixed on the effects of welfare-to-work programs on children [17,22,41,44,47].

Evaluation studies of welfare-to-work continue [48] but controversy remains [49-51] because of the many unintended consequences of these programs. Insights from recent studies reveal that single low-income parents face significant day-to-day challenges when integrating work and family demands, such as arranging child care [52]. Further, the transition from welfare to work emphasises economic issues (lack of health benefits) that are a root cause of welfare dependency [53].

### Obtaining health and social services

Since welfare-to-work initiatives, with or without income supplements, do not impact health in low-income families in a sustainable way, there is a need for interventions that are directly guided by the goal of enhancing health. The benefits of participating in health and social programs are well documented [46,54-58]. A consistent relation exists between family socioeconomic status and engagement/retention rate of families in health, social, educational, leisure, and cultural activities [7,8,59-61].

Barriers to the use of community-based health and recreation services cause low-income families to use primary health prevention and promotion services, including community resources and recreation, less often than other income groups [46,62-65]. For these families, personal barriers include (a) financial constraints, (b) under or unemployment, (c) mental and physical health problems, and (d) challenges to quality of life, such as communication difficulties associated with poor education and language barriers [66-70]. Families that overcome personal barriers often face barriers at the system level that include: fragmentation; narrowness of mandate; power differential created by provider expertise; and difficulty in access because of location, language, and hours of availability. Such service barriers lead to suboptimal outcomes and use of more expensive downstream societal resources.

In summary, low-income families face barriers to increasing their income and to obtaining a broad range of health and social services, thus negatively influencing their health and social outcomes. The evidence reviewed indicates that increasing access to service may be a direct and sustainable way to improve health and social outcomes in low-income families. An important goal for Canadian society must be to develop service-delivery policies and practices that reduce barriers to linkages between services and families in need, while being cost effective. This study focused on service integration approaches that actively link low-income families to healthy family lifestyle (e.g., health services, child care/schools, social supports) and to recreation.

### Families first Edmonton trial

In the Families First Edmonton (FFE) trial, we randomized 1168 low-income families into four service integration approaches to test proactive linkages between low-income families and services existing in their communities.

### Family healthy lifestyle and family recreation as service integration approaches

FFE was initiated after findings from When the Bough Breaks (WTBB), an award-winning Canadian study [24], were published. That study showed that that providing low-income families on social assistance with proactive comprehensive care (health promotion, employment retraining, and recreation activities for children) compared to allowing families be self-directed in finding these services, resulted in 15% more exits from social assistance within 1 year and substantial savings to society in terms of social assistance pay-outs.

Primary health care is the first element of continuing health and is designed to connect families to community services concerned with the broad determinants of health, including health services, education, and social support [71,72]. It is accessible, continuous, comprehensive, and coordinated, and is provided as closely as possible to where people live or work. Primary health care delivered through in-house family support has been shown to enhance both parents’ [13,73,74] and children’s well-being [73,75-78]. Social support accounts for a significant proportion of low-income family adaptation [79,80]. It is likely that parental involvement with childcare and schools leads to a richer family social support network [81-83]. More importantly, it has also been found that children whose families are linked to their schools have better academic achievement [83].

Regarding recreation, children who are active and engage in positive recreational opportunities benefit in physical well-being, psychosocial health (e.g., less anti-social behaviour), and resilience [13,14,84-89]. The lack of skill development programs for low-income children imposes short- and long-term negative consequences [69]. Children’s participation in recreation is also linked to positive impacts on family relationships [69,90] and parental adjustments that are related to positive child development outcomes [74,84,91].

The FFE investigation tested the findings and probed the conditions and generalizability of WTBB for current social assistance environments. FFE differs from WTBB in many ways. First, an analytic framework that describes the outcomes and intervening variables founded the research questions and plan for data analysis. Second, the primary health care and recreational service integration approaches and supporting practices were described in detail and closely audited to enable transfer of the successful service integration approach. Third, the sample aimed to be more inclusive to allow for examination of a broad socio-economic profile of low-income families, thus allowing researchers to address the question of who benefits the most and under what circumstances, questions typically neglected in this type of research [92]. WTBB included only single-parent families (98.1% female-headed) on social assistance. FFE included both single- and two-parent families. WTBB was conducted prior to the implementation of mandatory welfare-to-work initiatives. In Alberta, welfare-to-work initiatives require low-income families who receive income support to engage in employment or employment-related activities in exchange for income support once their youngest child is 12 months of age. Further, the sample from WTBB was predominately Euro-Canadian. FFE recruited participant families in an area where 54% of Edmonton’s Aboriginal population and 39% of Edmonton’s visible minorities reside, thus allowing for analysis based on these characteristics. Families who do not speak English were given the opportunity to participate. Fourth, the role of family functioning was explored as an intervening variable between the service delivery models and the primary outcome (family linkage to services) and to secondary outcomes (costs and health). Fifth, the service delivery models in WTBB were implemented for one year. In FFE, the models will be implemented for up to two years with follow-up for three years. This design facilitated examination of short-, intermediate-, and longer-term outcomes. Sixth, the service delivery models of FFE were designed by service delivery professional and researchers to be simple, transparent, easily tracked, and sustainable. Thus FFE was designed to generate the types of knowledge that will be essential if FFE interventions are to be adapted and implemented elsewhere.

### Principles of service integration practice

A systematic review of effective interventions for school-aged children, 29 reviews of 1102 intervention studies [93], drove the selection of practice principles for service integration practices within the content areas of Family Healthy Lifestyle and Family Recreation. The studied interventions encompassed a variety of program orientations including promoting physical and mental health, enhancing educational outcomes, fostering positive emotional adjustments, and preventing problem behaviours, suicide, and child abuse. Analysis showed that successful programs have seven characteristics. First, they were holistic and integrated. The complexity of the life of the child, parent, and family is addressed. Intervention strategies that target diverse areas (e.g., mental and physical health, safety, education, recreation, and youth employment) are needed. Second, successful programs resulted from collaborations that are multi-level and multi-sectoral. Third, successful programs were capacity building. They provide opportunities to build skill, rather than focusing exclusively on eliminating undesirable problems and behaviours. Fourth, successful programs were client-centred. For example, culturally appropriate services are offered and transportation barriers are addressed. Fifth, successful programs were community-based in what is available and situated in families’ neighbourhoods. Sixth, successful programs were long term, engaging at-risk children and youth long enough to show effects and enabling relationships between staff and participants to develop. Seventh, successful programs were well staffed with supportive personnel who are culturally similar to the population served. As a result, the practice principles selected to be embedded in the service integration content areas of Family Healthy Lifestyle and Family Recreation included family-centredness, cultural sensitivity, capacity building and reflection.

### Aim of the study

This study focused on evaluating service integration approaches directed at increasing linkages between low-income families and services existing in their communities. The primary research question was:

What are the effects of three community-based service-integration approaches (Family Healthy Lifestyle, Family Recreation and Comprehensive), as compared to a control model (Existing Services), on the number of linkages that families initiate and maintain with established health and social services?

The design of the study enabled a thorough examination of several additional questions that are critical for understanding the influence of different service-delivery models on family linkages with established services, costs, and health. Secondary research questions included:

1. What are the relative effects of different service integration approaches on specific characteristics of family linkages to established service (e.g., type of service, satisfaction with), and how do the frequency and type of involvement change over time?

2. What are the costs to service systems of each of the service-integration approach over time?

3. What are the physical and psychosocial health outcomes of family members, over time, associated with each of these service integration approaches?

In order to effectively contribute to policy change, it was important to identify concurrent and antecedent variables related to linkage, cost, and health outcomes, and to estimate the likely impact of these variables [94,95]. Tertiary research questions included:

4. What is the intervening role of family functioning (family problem solving, communication, parenting, connections to community) between each of the four service integration approaches and linkage to services, cost, and health outcomes for family members, and how does this role change over time?

5. To what extent does sociodemographic profile (e.g., ethnicity, immigrant status, education, occupation, family type, level of income, sources of income, security of housing, number of children) influence the relation between each of the four service integration approaches and linkage to service, cost, and health outcomes, and how does this influence change over time?

### Hypothesis

We hypothesized that low-income families who receive community-based service-integration (i.e., Family Healthy Lifestyle, Family Recreation and/or Comprehensive) would initially increase linkages to services, and that costs to service systems would decrease as improvements to physical and psychosocial health of family members are realized over time.

## Methods/Design

### Study design

FFE is a randomized, single-blind, longitudinal effectiveness trial with a 2 by 2 factorial design with three integration groups and a control group. The study was conducted in the City of Edmonton between January 2006 and August 2011. The study employed a multi-prong strategy to recruitment. Eligible families were invited to the study through five source programs for low-income families. In addition, posters and invitation packages were available at community agencies and through service workers. Information about the study was presented at community events where eligible families could inquire for more information. All families who received the source programs during the recruitment period were invited to participate in the study. Families were randomized after baseline data collection using a 1:1:1:1 allocation ratio. Those who were assigned to the intervention groups received between 18 and 24 months of service integration intervention. All families were followed by researchers for a total of three years with a minimum of ten interviews for data collection: two at baseline, and two at year 1, year 2, and year 3, respectively.

### Eligibility criteria for participants

The study focused on low-income families residing in City of Edmonton. To be eligible families needed to be receiving one of the five government assistance programs: (1) Income Support (“social assistance”), (2) Alberta Child Health Benefit, (3) City of Edmonton’s Leisure Access program, (4) Alberta Adult Health Benefit, and (5) Capital Regional Housing. All these programs provide either financial assistance or access to affordable housing and recreation for low-income individuals or families in the city area. In addition, potentially eligible families would have at least one child younger than 12 years of age living in the household. Families were allowed to define themselves: thus, the definition of family is not confined to traditional dual-parent family or biological-parent family but includes others such as single-parent families, adoptive families, and grandparent led families.

### Exclusion criteria

At recruitment, the study had three main exclusion criteria: (1) unwilling to commit to the full length of the study, (2) unwilling to provide researchers access to the focus child, or (3) non-English speaking families for whom researchers were unable to locate a relevant interpreter. After families were recruited, they were free to withdraw from the study at any time without penalty, with the understanding being that the intervention to which they were assigned was terminated. Although the study focused on low-income status, families with sufficient improvement in household income were not excluded from the study, due to recognition of income fluctuation in some vulnerable populations. After recruitment, families were no longer eligible to participate if they moved outside of the study area, the City of Edmonton.

### Details of the intervention

FFE community partners funded the practice content areas. The amount of direct service was constrained by two things: 1) the desire to evaluate the effect of a small intervention on the use of existing service and 2) the realities of the funders’ budget. For these reasons, Family Recreation was funded for 1.4 hours per month per participant family, Family Healthy Lifestyle was funded for 3.5 hours per month, and Comprehensive was funded for 4.6 hours per month.

The practice content areas (recreation and family healthy lifestyle), practice principles (family-centredness, cultural sensitivity, capacity building, and reflection) and hours of direct service were essential elements of logic model development followed by request for proposals (RFP). The logic model elements formed the basis of an FFE RFP from interested community agencies.

Existing service providers in Edmonton were invited to submit a proposal for delivery of the FFE interventions. Evaluation of the proposals for service delivery led to the selection of a service delivery partnership of four community agencies called Families Matter. The Families Matter partners included: YMCA of Edmonton, Multicultural Health Brokers Co-operative, KARA Family Resource Centre, and Bent Arrow Traditional Healing Society. To achieve practice rigor Families Matter relied on: hiring practices that target selected front-line provider education and experience; in-service training directed at developing knowledge and behaviours believed to match the practice principles and content area selected; and supervision approaches.

Community-based intervention, when delivered in a research project, risks losing intervention fidelity for at least two reasons: (a) use of general practice principles and very broadly identified content area within which to practice, and (b) intervention drift [96]. In addition, there is a culturally based reluctance by service providers to submit to rigorous oversight of community developed practices.

For these reasons, action research methods were used to record and monitor the delivery of the service integration approaches. An administrative database was jointly developed to include qualitative and quantitative methods of recording practice to be used to calculate dose and to audit practices. In addition, the administrative and supervisory staff of Families Matter met weekly with the researchers to review and internalize the elements of the FFE service delivery logic model, which built the relationship and internalized the need for intervention fidelity. Similarly, a researcher spent half a day each week with the supervisors and family workers, focussing on trouble-shooting the practices associated with recording the practices in the data base and on the need for fidelity to the three service integration groups. Families Matter also assigned family workers and supervisors to only one service integration approach in order to support intervention fidelity. Lastly, focus groups and individual interviews were held, with supervisors and family workers, over the course of the 18 months of service delivery in order to specify the practices used in service integration. At the end of the project, a Service Integration Tool Kit was published [97].

### Outcome measures

The study aimed to determine which of the interventions impact accessibility in the most efficient and effective manner. Where possible, the measures were selected because of their standardization, the presence of published norming data, and their utility as comparators to other studies of low-income families.

The primary outcome, family linkage to health and social services, was measured using an innovative tool, “Family Services Inventory” (FSI). This tool was developed by a health economist, in consultation with FFE researchers. The FSI adopts a societal perspective and measures public and private consequences for families. The FSI was developed to maximize precision while minimizing patient burden and recall bias. A FSI toolkit comprised in-service training materials for data collectors, a user manual, a codebook, and a calendar (a memory aid to reduce recall bias). Annual data capture of resource use during the prior month was conducted in face-to-face interviews. Due to variations in scheduling interviews, resource use is normalized to the observation period (time between first and second visit). Characteristics of services and data allowing for costing of services were also collected in the FSI.

An overview of measures associated with the primary, secondary and intervening outcomes is found in Table 1.

**Table 1:** Outcomes, measures, and instruments

**Outcome**	**Measure**	**Instrument**
	**€ Primary Outcomes €**	
Linkages between low-income families and established services in their communities	Average number of times the family linked to services	Family Services Inventory (FSI)
Quality of life	Preference based measures	Visual Analogue Scale (EQ VAS) Australian Quality of Life (AQoL)
	**€ Secondary Outcomes €**	
Characteristics of linkages between families and services	Type of service, satisfaction	FSI
Costs to service systems	Costs of services linked to	FSI
Adult physical health	General health status	Single general health item from National Longitudinal Survey of Children and Youth (NLSCY), EQ-5D
Adult psychosocial health	Somatization, obsessive-compulsive, interpersonal sensitivity, depression, anxiety, hostility, phobic anxiety, paranoid ideation, psychoticism, self-esteem	Symptom Checklist (SCL-90-R), Self-Esteem Inventory
Linkages to community	(a) Perceived social functions and provisions obtained from relationships with others; (b) social and civic participation; (c) barriers to participation; (d) neighbourliness	(a) Social Provision Scale; (b) items from the Health and Participation survey; (c) Left Out survey; (d) items from NLSCY
Child physical health	General health status, chronic conditions, injuries, nutrition, sleep pattern, risk behavior	40 items from NLSCY
Child psychosocial health	Socio-emotional development, personality, behavioral problems, attitudes, mental health	Behavioral Assessment System for Children (BASC)
Child Achievement	Spelling, Arithmetic, Reading, Receptive Vocabulary	Wide Range Achievement Test; Peabody Pictorial Vocabulary III
Family Functioning	(a) Problem solving & communication skills, (b) parenting,	McMaster Family Assessment device
Socio-demographics	Ethnicity, immigration status, education, occupation, family type, level of income, source of income, security of housing, number of children, neighbourhood	Items from NLSCY

### Data collection and management

Data were collected at baseline, prior to randomization, and then every 12 months for three years. One adult respondent per family was selected based on familiarity with the children. A focus child was randomly selected (using computer-generated randomized lists stratified by number of eligible children) amongst the children within the household at screening. When appropriate, children’s data were collected from the focus child. During data collection phase, follow-up data collections were conducted annually for all families. Research assistants who had established rapport with families since first contact arranged each data collection. Data collectors, blind to the group assignment, collected data during home visits. FFE researchers adopted a professional human resource model with a data collection supervisor who oversaw 29 data collectors and 36 interpreters. We adopted this model because (a) a number of our instruments are standardized measures requiring psychometric assessment skills, and (b) it enhanced our ability to attract and retain a staff with adequate credentials and experience. Most of our data collectors had completed or were working toward their Master’s degrees in a variety of health and social science fields. Data collectors were required to work evening and weekends, to be responsive to the schedules of the families, and all were rigorously trained on the data collection process, crisis management, and cultural sensitivity. A detailed manual of operations was available to the research assistants and data collectors at the coordinating office. Due to the volume of the data collected and to avoid fatigue of the primary participants, each data collection point consisted of two visits approximately 28 days apart. The 28 days lapse also allowed for a prospective collection of the services accessed by the family.

All data were collected using standardized case report forms by trained data collectors and then faxed to the Epidemiology Coordinating and Research (EPICORE) Centre -- a clinical trials and health services research centre within University of Alberta, for data entry. EPICORE was responsible for data storage and quality assurance with the help of the data collection team. The database was coded in Microsoft Visual FoxPro V7.0. The use of free text fields and open-ended questions were eliminated from the forms, wherever possible. Simple (e.g., aberrant values, missing values) and complex verification rules were applied. Study subjects were identified by a unique study number assigned at time of enrolment. The study participants’ personal information was not included in the database. Confidential contact information and documents containing the link between the primary parent’s and focus child’s names and the assigned study number were stored in locked file cabinets at the FFE coordinating office. Sixty randomly sampled from the first 200 baseline data files (all forms in part 1 and 2) were audited. The transcription error rate was less than 1% (5 errors were identified).

### Randomization

The random allocation sequence was computer generated using R version 2.8.1 software.

We used permuted blocks of 8 and 12, stratified by type of income (Income Support receipts versus other source programs) and age of the focus child (0 to 3.9, 4 to 6.9, 7 to 9.9, 10 to 12.9 years).

### Allocation to experimental group and implementation

The intervention assignments were concealed on serially numbered, opaque, sealed envelopes. After the baseline data collection was completed, research assistants phoned the families and then opened randomization envelopes that were pre-stratified according to low-income status (i.e., receiving income supports vs. other source programs) and age of the Focus Child. Families were informed of their group assignment at that time. Research assistants explained the next steps to families. For those allotted to the intervention groups, their contact information was passed onto the service delivery workers. Research assistants explained to families that researchers would be in contact again for follow-ups, but families could contact them before then if they were moving or had any concerns about the study.

### Blinding

The data collectors and the investigators were blinded from the group assignments. The families, the service delivery workers and the research assistants were not blind to the group assignments at any point in the trial. Investigators were unblinded when the datasets were cleaned (January 2012).

### Sample size

Projecting a moderate (f = 0.25) and even a small (f = 0.10) intervention effect size (mean divided by standard deviation), given an alpha of 0.05 and a 25% attrition rate, the study proposed an initial sample size of 300 families per group to detect any significant difference between the four groups (power was 0.99 and 0.72, respectively). The study randomized approximately 290 families to each group. The overall alpha value was not controlled for multiple comparisons. No interim analyses were planned.

### Statistical methods

Most analyses will follow the intent-to-treat principle. The last-observation-carried-forward method will be used to impute missing data to maintain the integrity of the data set. Data will be analyzed using Stata MP 13.0 and SPSS 21.

### Primary analysis

The data will be analyzed using linear regression including terms for the healthy family lifestyle and family recreation service-integration approaches, their interaction and, where appropriate, time (a repeated measure). If the distribution of the primary outcome is right-skewed and cannot be transformed to a normal distribution, Poisson regression will be used. Estimates and corresponding 95% confidence intervals will be reported. The income group (Income Support vs. other source program) and the age of the focus child corresponding to strata variables in randomization will be included in the model.

Analysis of the FSI and quality of life data will be guided by the Canadian guidelines for the economic evaluation of health technologies [98], which comprises a five-part analysis. Part 1 will focus on identifying and describing the differences in use of social and health system services. Part 2 will result in the translation of these differences into dollar values, including the valuation of the intervention. Part 3 will focus on identifying and measuring the differences in quality of life for the children, parents and families. Part 4 will integrate the costing and quality of life information into a formal cost-utility analysis. Beginning with a budget impact analysis, Part 5 will examine the implications of the findings from an administrative and policy perspective.

### Other analyses

Additional variables to be considered in exploratory analyses include ethnicity, immigrant status, education, occupation, family type (e.g., single-parent families), level of income, sources of income (e.g., part-time), security of housing, number of children, region of the city. Residual, leverage, and influence diagnostics will be examined. For secondary outcomes, the four groups will be analyzed using appropriate modelling, such as linear regression, when the data are normal and logistic regression when the data are dichotomous. To study relations between family functioning and interventions over time, latent growth curve analysis will be used [99]. This method provides a comprehensive framework for studying latent variables over time by combining the strengths of structural equation modelling. This approach will enable modelling of the interaction among key variables, with the statistical techniques associated with longitudinal data analysis, which will allow us to study how these variables and their relations change over time. To exploit the power of latent growth curve analysis we will first develop and test theoretically derived and empirically based models designed to link variables using structural equation modelling methods at one point in time. Once we have developed and tested our initial models, we will extend our analyses by adding a growth parameter to each model so we can evaluate change over many periods in time. This combination of modelling variables within and between time periods has the potential to increase our understanding of variation while expanding the scope and value of the study.

### Ethical considerations

This study protocol was approved by a special interdisciplinary ethics committee, led by the Faculty of Education, University of Alberta, Edmonton, Canada and Director (file number Pro00000144) of the then newly formed Human Research Protections Office. Ethical review for subsequent changes in the protocol, which are described below, were reviewed and approved. Although this is a community-based randomized controlled trial (RCT), partners do not have access to the original data and researchers do not receive any salary or other compensation during the trial.

Interested families contacted the community-based research office to learn about the study and ask questions. Families in which English was a second language were offered the option of an interpreter during all interactions (e.g., on the phone setting up appointments, during the times the study was explained, during data collection). At the first home visit, each family was given written information about the study, which the data collector reviewed verbally. Written informed consent was obtained prior to study entry and data collection. Families were given an honorarium at each data collection period ($25 in early data collection periods and $30 at the final data collection period).

## Discussion

Modifications were made based on the pragmatics of community-based trials.

### Recruitment

The investigators intended to recruit families in six months with three main eligibility criteria. It was planned that families must (a) have received one of the two source provincial programs, either Income Support or Alberta Child Health Benefits, continuously for the past six months, (b) reside in the central and North-east quadrants of the City of Edmonton, and (c) have at least one child younger than 12 years of age. The number of families in the sampling frame that met the original criteria was estimated to be about 5000. In fact, within six months of recruitment it was apparent that the target would not be reached. The actual response rate using these eligibility criteria was about 10%.

The research team reviewed the recruitment process and eligibility criteria, and concluded: (1) all currently eligible families had already been invited; (2) the original eligibility criteria excluded many low-income families who were not currently on the two pre-identified source programs. Many immigrants or those on federally funded aboriginal treaty programs were not entitled to or were unaware of the two provincial programs. In addition, some eligible families were opting not to apply for these programs.

To increase the recruitment number and to ensure a representative sample of low-income families, the eligibility criteria were relaxed and extended: (1) the requirement that families had to have been receiving the source program for at least 6 months was waived; (2) source programs were added and (3) recruitment was expanded from central and northeast to the whole City of Edmonton (pop. ≈ 800,000).

### Duration of intervention and follow-up data collection schedule

As a result of expanding the recruitment period to 24 months, those enrolled during the last 6 months of recruitment only received up to 18 months of services rather than 24 months. Originally FFE was planned to be a five-year research project with eight visits for eight data collection points (baseline, 6 mos., 12 mos., 18 mos., 24 mos., 36 mos., 48 mos., 60 mos.). To increase the accuracy of the service utilization data, the research team adopted a prospective approach resulting in having two visits for each data point (16 home visits). To contain costs, the FFE Steering Committee in consultation with the research team approved dropping four data points: 6 mos., 18 mos., 48 mos., and 60 mos. That is, the FFE trial collected data from each family once a year for three years but data collection activity was spread over five years (accounting for the expanded recruitment period).

### Participant flow

**Figure 1:**
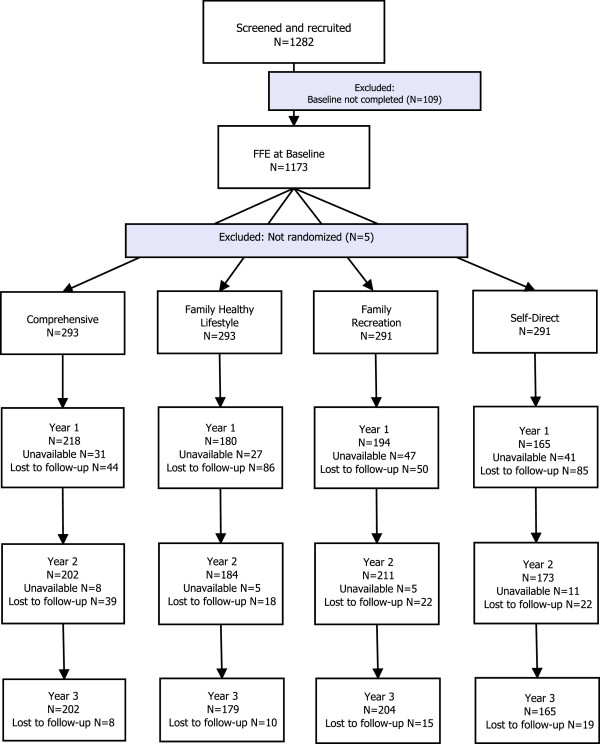
Participant flow.

Figure 1 describes, the number of participants successfully screened and recruited and, for each group, the number of participants who were randomly assigned and received intended treatment.

Of the families who were contacted and expressed interests in the study, 1282 families were successfully screened and met recruitment criteria. Because some families withdrew and contact with others was lost, only 1168 families were randomized into the four groups.

### Losses and exclusions after randomization

As indicated in Figure 1, during the process participants could either withdraw (i.e., lost to follow-up) or be ‘dormant’ for some of the data collection points (i.e. unavailable). At 36 months we were able to collect data from 748 families; data from 414 families were lost (135 Existing Services, 88 Family Recreation, 110 Family Healthy Lifestyle, and 91 Comprehensive). Table 2 shows reported reasons for withdrawal.

**Table 2:** Losses and exclusions

**Reasons for withdrawal**	**Before randomized**	**After randomized**	**Reasons for withdraw (count)**
Not interest in	55	18	73
Too busy/No time	47	40	87
Move out of study area	10	88	98
Family circumstances	8	9	17
Problems with research	6	4	10
Problems with randomized design	5	-	23
Problems with services allotted	-	18	18
Health	3	5	8
Kids not interests in	3	1	4
Too much time past since last contact	3	0	3
Cultural differences	1	0	1
Stigmatization	0	1	1
Unknown	18	5	23
**Grand Total**	**159**	**189**	**366**

## Abbreviations

CUP: Community-university partnership for the study of children, youth, and families; EPICORE: Epidemiology coordinating and research centre; EQ VAS: EQ visual analogue scale; FFE: Families first edmonton trial; FSI: Family services inventory; GSRM: Governments of Canada strategic reference models; NLSCY: National longitudinal study of children and youth; RCT: Randomized controlled trial; RFP: Request for proposal; WTBB: When the bough breaks.

## Competing interests

KF and NW are consultants to the team. Notwithstanding, the authors declare that they have no competing interests and are not and have not been employed by the funding bodies that include: Alberta Heritage Foundation for Medical Research (AHFMR), and Canadian Institute for Health Research (CIHR), Canadian Health Services Research Foundation (CHSRF).

## Authors’ contributions

The authors all played significant roles in the study. JD (Principal Investigator), LS (Research Director, RCT), SS (Research Coordinator, RCT), MM (Research Director, Collaboration), DW (Lead, Family Data Analysis), JB (Lead, Child Data Analysis), KF (Lead, Service and Economic Analysis), and NW (Bio-statistician) have all been part of the development, implementation and analyses. JD, LS, and SS drafted this manuscript; all authors provided comments on the drafts and have read and approved the final version.

## Authors information

Randomized controlled trials are rare in community-based intervention research and demand an interdisciplinary team that includes researchers with strong content knowledge, methodological skills, and collaborative nature. FFE managed by the Community-University Partnership for the Study of Children, Youth, and Families (CUP), an umbrella organization co-owned by academic, government, and community leaders. In July, 2000, CUP was formed to promote reciprocal, sustained, and mutually beneficial interactions among researchers, practitioners, and policymakers in research, education, and knowledge mobilization. JD, LS, MM, JB, DW all played leadership roles in CUP on and off campus. For more information, please visit http://www.cup.ualberta.ca or http://www.familiesfirstedmonton.ualberta.ca/.

## Pre-publication history

The pre-publication history for this paper can be accessed here:

http://www.biomedcentral.com/1472-6963/14/223/prepub
